# p.Val804Met, the Most Frequent Pathogenic Mutation in *RET*, Confers a Very Low Lifetime Risk of Medullary Thyroid Cancer

**DOI:** 10.1210/jc.2017-02529

**Published:** 2018-03-23

**Authors:** Chey Loveday, Katherine Josephs, Daniel Chubb, Adam Gunning, Louise Izatt, Marc Tischkowitz, Sian Ellard, Clare Turnbull

**Affiliations:** 1 Division of Genetics and Epidemiology, The Institute of Cancer Research, London, United Kingdom; 2 Department of Clinical Genetics, St George’s University Hospital, London, United Kingdom; 3 Institute of Biomedical and Clinical Science, University of Exeter Medical School, Exeter, United Kingdom; 4 Department of Clinical Genetics, Guy’s and St Thomas’ NHS Foundation Trust, London, United Kingdom; 5 Department of Medical Genetics, University of Cambridge, Cambridge, United Kingdom; 6 William Harvey Research Institute, Queen Mary University, London, United Kingdom; 7 National Cancer Registration and Analysis Service, Public Health England, London, United Kingdom

## Abstract

**Context:**

To date, penetrance figures for medullary thyroid cancer (MTC) for variants in rearranged during transfection (*RET*) have been estimated from families ascertained because of the presence of MTC.

**Objective:**

To gain estimates of penetrance, unbiased by ascertainment, we analyzed 61 *RET* mutations assigned as disease causing by the American Thyroid Association (ATA) in population whole-exome sequencing data.

**Design:**

For the 61 *RET* mutations, we used analyses of the observed allele frequencies in ∼51,000 individuals from the Exome Aggregation Consortium (ExAC) database that were not contributed via The Cancer Genome Atlas (TCGA; non-TCGA ExAC), assuming lifetime penetrance for MTC of 90%, 50%, and unbounded.

**Setting:**

Population-based.

**Results:**

Ten of 61 ATA disease-causing *RET* mutations were present in the non-TCGA ExAC population with observed frequency consistent with penetrance for MTC of >90%. For p.Val804Met, the lifetime penetrance for MTC, estimated from the allele frequency observed, was 4% [95% confidence interval (CI), 0.9% to 8%].

**Conclusions:**

Based on penetrance analysis in carrier relatives of p.Val804Met-positive cases of MTC, p.Val804Met is currently understood to have high-lifetime penetrance for MTC (87% by age 70), albeit of later onset of MTC than other *RET* mutations. Given our unbiased estimate of penetrance for *RET* p.Val804Met of 4% (95% CI, 0.9% to 8%), the current recommendation by the ATA of prophylactic thyroidectomy as standard for all *RET* mutation carriers is likely inappropriate.

## Teaser

We used population data to estimate unbiasedly penetrance of pathogenic RET mutations for medullary thyroid cancer, revealing that p.Val804Met is vastly less penetrant than other RET mutations at ∼4%.

Multiple endocrine neoplasia type 2 (MEN2) was first recognized in the mid- to late-1960s as a pleomorphic cancer-susceptibility syndrome, characterized by a high risk of medullary thyroid cancer (MTC) (1–3). Clinically distinct subtypes of MEN2 were subsequently described with MEN2A, characterized by MTC plus pheochromocytoma and parathyroid disease; MEN2B featuring MTC plus marfanoid habitus, benign oral, and submucosal tumors; and familial MTC (FMTC) with MTC alone. In 1993, the rearranged during transfection (*RET*) protooncogene was definitively identified as the gene underlying MEN2 (4, 5), and germline mutations have subsequently been detected in nearly all families with MEN2A, MEN2B, and FMTC, with emergence of a distinct pattern of genotype-phenotype correlation (Table 1). More recently, the American Thyroid Association (ATA) categorized *RET* mutations based on age-related penetrance of MTC. The 2009 ATA guidance adopted classes A through D (with D as the most severe); in the 2015 revisions, they were updated to “moderate”, “high” and “highest” risk of MTC, with accordant guidance on timing of prophylactic thyroidectomy for unaffected *RET* mutation carriers (Table 1) (6, 7).

**Table 1.** Genotype-Phenotype Correlations in MEN2

ATA Risk Level ^*a*^	*RET* Mutations ^*b*^	MEN2 Subtype	Age of MTC	ATA Recommendations for Thyroidectomy ^*a*^
Moderate (ATA-MOD)	p.Gly533Cys; p.Lys603Glu; p.Tyr606Cys; p.Cys609Arg/Gly/Phe/Ser/Tyr; p.Cys611Arg/Gly/Phe/Ser/Trp/Tyr; p.Cys618Arg/Gly/Phe/Ser/Tyr; p.Cys620Arg/Gly/Phe/Ser/Trp/Tyr; p.Cys630Arg/Phe/Ser/Tyr; p.Asp631Tyr; p.Lys666Glu; p.Glu768Asp; p.Asn777Ser; p.Leu790Phe; p.Val804Leu/Met; p.Arg833Cys; p.Arg844Gln; p.Ser891Ala; p.Arg912Pro	FMTC and MEN2A	>5 y	In childhood (by 5 or 10 y) or adulthood or when serum calcitonin level becomes elevated
High (ATA-H)	p.Cys634Arg/Gly/Phe/Ser/Trp/Tyr; p.Ala883Phe	MEN2A	<5 y	At or before 5 y, based on calcitonin serum levels
Highest (ATA-HST)	p.Met918Thr	MEN2B	<1 y	First y or first mos of life

Abbreviation: ATA, American Thyroid Association.

^a^ As per the revised ATA 2015 guidelines (7).

^b^ Mutations annotated using Reference Sequence accession NM_020975.

As typical for rare Mendelian disease, studies correlating mutational frequencies of *RET* with disease manifestation of MTC/MEN2 are based on clinical case series, ascertainment of which is necessarily based on their phenotype. Only through the studying of a population ascertained agnostic to phenotype can unbiased estimates of disease penetrance for a given variant be calculated. Whereas data from large-scale sequencing of “true” population controls are not yet available, unbiased estimates of cancer penetrance can be derived from sequencing data aggregated from cohorts of individuals with “common complex” conditions unrelated to cancer, such as hypertension, autoimmune disease, and diabetes (8–11). Here, we present analysis of “population” sequencing data from ∼51,000 individuals for the 61 *RET* mutations designated as disease causing by the ATA.

## Materials and Methods

### Identification of pathogenic *RET* mutations

RET protein alterations, designated as disease causing (pathogenic) by the ATA, were identified within the published guidelines, and matching variants were retrieved from ClinVar, along with their corresponding changes at the nucleotide level (6, 7, 12, 13). We identified a total of 61 different pathogenic *RET* single nucleotide variants, corresponding to 53 different protein alterations at 24 different residues (Tables 1 and 2).

**Table 2.** *RET* Mutation Allele Counts in Non-TCGA ExAC

*RET* Mutation^*a*^	Non-TCGA ExAC^*b*^			90% Penetrance		50% Penetrance	
	Allele Count	Alleles Observed	Minor Allele Frequency	Alleles Expected^*c*^	*P* Value^*d*^	Alleles Expected^*c*^	*P* Value^*d*^
ATA moderate-risk level (MOD)							
c.1799G > A:p.Arg600Gln	80,494	3	3.73 × 10^−5^	2	4.79 × 10^−1^	3	1.00 × 10°
c.2330A > G:p.Asn777Ser	105,944	1	9.44 × 10^−6^	3	2.48 × 10^−1^	4	1.34 × 10^−1^
c.2370G > C:p.Leu790Phe	105,414	1	9.49 × 10^−6^	3	2.48 × 10^−1^	4	1.34 × 10^−1^
c.2370G > T:p.Leu790Phe	105,414	2	1.90 × 10^−5^	3	5.64 × 10^−1^	4	3.17 × 10^−1^
c.2410G > A:p.Val804Met	55,798	11	1.97 × 10^−4^	2	1.96 × 10^−10^	3	3.86 × 10^−6^
c.2410G > T:p.Val804Leu	55,798	1	1.79 × 10^−5^	2	4.79 × 10^−1^	3	2.48 × 10^−1^
c.2497C > T:p.Arg833Cys	104,240	2	1.92 × 10^−5^	3	5.64 × 10^−1^	4	3.17 × 10^−1^
c.2531G > A:p.Arg844Gln	105,686	4	3.78 × 10^−5^	3	5.64 × 10^−1^	4	1.00 × 10°
c.2735G > C:p.Arg912Pro	106,202	1	9.42 × 10^−6^	3	2.48 × 10^−1^	4	1.34 × 10^−1^
ATA high-risk level (H)							
c.1900T > C:p.Cys634Arg	106,030	1	9.43 × 10^−6^	3	2.48 × 10^−1^	4	1.34 × 10^−1^
c.1901G > T:p.Cys634Phe	106,036	1	9.43 × 10^−6^	3	2.48 × 10^−1^	4	1.34 × 10^−1^

^a^ Fifty ATA pathogenic *RET* single nucleotide variants, not observed in non-The Cancer Genome Atlas Exome Aggregation Consortium (TCGA ExAC), collapsed by residue: moderate-risk level: p.G533C, p.K603E, p.T606C, p.C609S/R/G/T/F, p.C611S/R/G/T/F/W, p.C618S/R/G/T/F/W, p.C620S/R/G/T/F/W, p.C630S/R/T/F, p.D631T p.K666E, p.E768D, p.E768D, p.Val804L, p.S891A; high-risk level: p.C634S/G/T/W, p.A883F; highest-risk level: p.E805K, p.T806C, p.M918T. Annotated using Reference Sequence accession NM_020975.

^b^ Non-TCGA ExAC data for ∼51,000 individuals. The age of individuals included in this dataset, at the time of ascertainment, ranges from 18 to 85, with most individuals aged 40 to 70 y.

^c^ Calculated using the method of Whiffin *et al.* (14). MTC lifetime risk estimated at one in 3000, genetic heterogeneity is estimated at 20%, allelic heterogeneity at 25%, and powered for penetrance >90% and >50%.

^d^ One-way *χ*^2^ test to compare observed and expected values using a Bonferroni corrected *P* value threshold for significance for performing 61 tests (*P* value threshold = 8.2 × 10^−5^).

### Population dataset

We used a subsetted version of the Exome Aggregation Consortium (ExAC) database that had excluded individuals from The Cancer Genome Atlas (TGCA; non-TCGA ExAC; release 0.3.1) to obtain estimates of the frequency of pathogenic *RET* mutations in common complex disease cohorts not related to cancer (8). This subset of ExAC comprises ∼51,000 individuals, the vast majority of which are aged between 40 and 70 years at the time of ascertainment (10), with a male-to-female ratio of 1.3:1.

### Calculation of “expected” allele counts

With the use of the methods of Whiffin *et al.* (14), we calculated the maximum number of mutant alleles that we would expect to observe in the non-TCGA ExAC data for a pathogenic *RET* mutation. In brief, this method leverages the logic that a fully penetrant allele cannot be more common than the disease it causes. Therefore, for a given disease/gene, the maximum credible population allele frequency can be calculated by the multiplication of lifetime risk (expressed as one in *n*) by total allelic contribution (expressed as a value between zero and one), divided by penetrance (expressed as a value between zero and one), multiplied by two (reflecting the diploid human genome). The maximum tolerated allele count is then determined based on the population size at a given level of confidence. When assigning a value to each parameter used in the model, we selected the most conservative estimate of the range represented in the literature, *i.e.*, the highest estimates for lifetime risk and total allelic contribution and the lowest estimate for penetrance. The application of values for lifetime risk of disease and total allelic contribution that are higher than the true underlying values will overestimate the expected allele count. Likewise, the underestimation of penetrance will also overestimate the expected allele count. *P* values were calculated for observed vs expected values using a one-way *χ*^2^ test. The *P* value threshold for significance was corrected for performing 61 tests (*P* value threshold = 8.2 × 10^−5^). We define in the following sections how the parameters modeled to calculate expected allele counts for application were derived.

#### Lifetime risk

The reported lifetime risk of MTC varies widely: Orphanet Encyclopedia suggests a lifetime risk of 1:14,300 (15). Reported incidence of MTC varies by sex and population. The two largest populations in ExAC are the following: (1) Utah residents with Northern and Western European ancestry and (2) British in England and Scotland, which comprise 24% and 17% of total individuals in ExAC, respectively (10). In the United States, the lifetime risk of thyroid cancer is estimated to be one in 167 men and one in 56 women, with 1% to 2% comprising the medullary subtype (7). With the use of the upper bound of these estimates, this equates to a lifetime risk of MTC in the United States of one in ∼8300 for men and one in ∼2700 for women (one in ∼4100 overall). In the United Kingdom, the lifetime risk of thyroid cancer is estimated to be one in 480 in men and one in 180 in females (16), with ≤8% of the medullary subtype (17, 18). With the use of the upper bound of these estimates, this equates to a lifetime risk of MTC in the United Kingdom of one in 6000 in men and one in 2250 in women (approximately one in 3300 overall). For the purpose of this study, we use a conservatively high (common) estimate of one in 3000 for the risk of MTC across a UK/US population of Northern and Western European ancestry [which is far higher and therefore, more conservative than the underlying frequency of 1:14,300, suggested by the Orphanet Encyclopedia (15)]. We have also not corrected for the sex disparity in ExAC (which would equate to a predicted lower average lifetime risk as a result of the higher proportion of men to women in this cohort).

#### Total allelic contribution

Total allelic contribution is a function of both genetic and allelic heterogeneity. Genetic heterogeneity refers to the maximum proportion of a disease attributable to a single gene, whereas allelic heterogeneity refers to the maximum proportion of pathogenic variation within a gene attributable to a specific single allele. We used data from the largest recent published dataset from systematic data collection from a 20-year Italian single center experience of *RET* testing, which included 1556 subjects (19). In this series, ∼8% of MTC cases have a family history of MTC (with *RET* mutations detected in 98% of multiplex MTC families), and *RET* mutations are detected in an additional ∼7% of “seemingly” sporadic MTC cases (19, 20). With the use of an upper range of the sum of these two elements, we apply to the model a conservatively high estimate for genetic heterogeneity of 0.20.

In this series, the most frequently detected mutation was seen in 31/139 probands (22.3%, p.Val804Met), with the second-most populous pathogenic mutation detected in 17/139, (12.2%, p.Cys634Arg) (19). With the use of an upper range on these estimates, we defined the maximum allelic heterogeneity for a pathogenic mutation in *RET* to be 0.25. Thus, the highest total allelic contribution for a single *RET* mutation to MTC is 0.05 (0.25 × 0.20).

#### Penetrance

Most literature on pathogenic germline *RET* mutations describes near-complete lifetime penetrance for MTC, with the assumption only of differences in age-related penetrance for mutations in different ATA subgroups (7). To explore penetrance for pathogenic germline *RET* mutations in this study, expected allele counts were calculated under two models: (1) the first model assuming near-complete lifetime penetrance (90%) and (2) a second model assuming reduced lifetime penetrance (50%).

### Calculating penetrance for *RET* p.Val804Met

**Figure 1.**
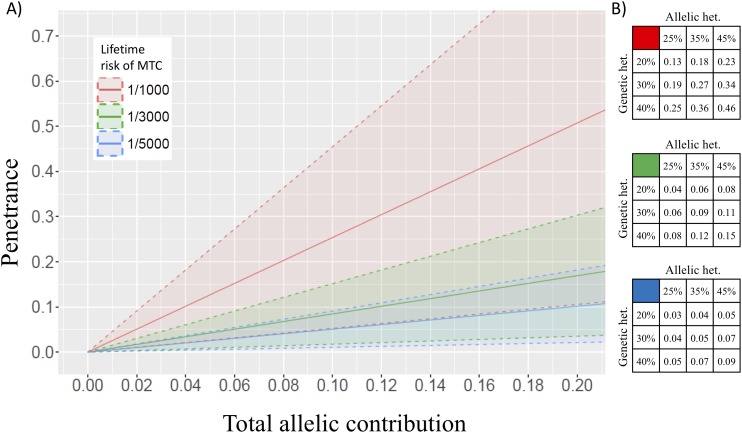
Penetrance for *RET* p.Val804Met as a function of genetic heterogeneity, allelic heterogeneity, and lifetime risk of MTC. (A) Total allelic contribution (*x*-axis) is the product of the genetic heterogeneity, multiplied by allelic heterogeneity [*e.g.*, a genetic heterogeneity of 20% and an allelic heterogeneity of 25% gives a total allelic contribution of 5% (or 0.05)]. Penetrance estimates were generated using lifetime risk under three scenarios: a lifetime risk, one in 3000 [a conservative estimate derived from a lifetime risk of MTC in the United States/United Kingdom (green line) and sensitivity testing using lower and higher estimates: one in 5000 (blue line) and one in 1000 (red line). Dashed lines and shading indicate 95% confidence intervals (CIs). (B) Point estimates of penetrance are shown by lifetime risk of MTC of one in 3000 (green fill), one in 5000 (blue fill), one in 1000 (red fill) with literature-derived estimates for genetic heterogeneity (25%), and allelic heterogeneity (20%) and sensitivity testing using higher estimates (35% and 45%, 30% and 40%, respectively).

We went on to explore penetrance for *RET* p.Val804Met, using the same model but fixing the allele count observed in non-TCGA ExAC as the maximum credible population allele frequency. We first applied our best baseline values derived from the literature for lifetime risk of MTC (one in 3000) and total allelic contribution (0.20 × 0.25) to generate the best estimate of penetrance for *RET* p.Val804Met compatible with the allele count observed in non-TCGA ExAC. We went on to perform sensitivity testing on this penetrance estimate by applying different estimates of lifetime risk of MTC (one in 1000, one in 3000, one in 5000), genetic heterogeneity (0.20, 0.30, 0.40), and allelic heterogeneity (0.25, 0.35, and 0.45; Fig. 1), generating 95% confidence intervals (CIs) based on the observed allele frequency.

## Results

### Frequency of pathogenic *RET* mutations in non-TCGA ExAC

Of the 61 pathogenic *RET* mutations documented in the ATA guidelines (Table 1), 11 were present in individuals in the non-TCGA ExAC population cohort. Fifty of the 61 mutations were not detected at all in this population cohort. Of the 11 pathogenic *RET* mutations present in individuals in non-TCGA ExAC, nine were in the moderate risk category, and two were in the high risk category (Table 2).

With the use of the conservative baseline estimates from the literature for model parameters (MTC lifetime risk: one in 3000, genetic heterogeneity: 20%, allelic heterogeneity: 25%), only c.2410G > A p.Val804Met had an observed allele count that significantly exceeded that which would be expected if the penetrance for MTC for this mutation was ≥90%, (Table 2; observed allele count = 11, expected allele count = 2, *P* = 1.96 × 10^−10^). The difference between observed and expected allele counts for p.Val804Met remained significant, even when applying a model based on a substantially reduced penetrance of 50% (expected allele count = 3, *P* = 3.86 × 10^−6^). Of note, the minor allele frequency (MAF) for p.Val804Met was consistent across the four main subpopulations constituting the non-TCGA ExAC: non-Finnish Europeans MAF = 0.000175 [observed (O) = 5, expected (E) = 2, *P* = 3.39 × 10^−2^]; South Asian MAF = 0.000260 (O = 3, E = 1 *P* = 4.55 × 10^−2^); Latino MAF = 0.000358 (O = 2, E = 0); and East Asian MAF = 0.00027 (O = 1, E = 0; Table 2). The MAF for p.Val804Met in the larger Genome Aggregation Database (MAF = 0.00014, from 129,886 individuals) was consistent with that in the non-TCGA ExAC (MAF = 0.0002).

Observed allele counts for all 60 other pathogenic *RET* mutations were consistent with our baseline model, assuming a penetrance ≥90%.

### Estimates for penetrance of p.Val804Met

With the use of the conservative, best baseline estimates from the literature for model parameters (MTC lifetime risk: one in 3000, genetic heterogeneity: 20%, allelic heterogeneity: 25%), the observed frequency of p.Val804Met in non-TCGA ExAC is consistent with a lifetime penetrance of MTC of 4% (95% CI, 0.9% to 8%). In sensitivity testing, the application of parameter estimates hugely exceeding those consistent with existing data (population incidence: one in 1000, genetic heterogeneity: 40%, allelic heterogeneity: 45%), the population frequency observed was consistent with a penetrance of only 46% (95% CI, 1% to 82%; Fig. 1).

## Discussion

Here, we present population data for p.Val804Met that shows a frequency greater than fivefold higher than that of any other pathogenic *RET* mutation. For all other pathogenic *RET* mutations, the observed frequency is consistent with a lifetime penetrance of MTC of >90%, whereas for *RET* p.Val804Met, the observed frequency is not even consistent with a lifetime penetrance of 50% (*P* = 3.86 × 10^−6^; Table 1). We go on to demonstrate that the observed frequency of p.Val804Met in non-TCGA ExAC supports a lifetime penetrance of MTC for p.Val804Met of 4% (95% CI, 0.9% to 8%). Even allowing for a much higher lifetime risk of MTC, *e.g.*, one in 1000, the observed frequency is only compatible with a lifetime penetrance for MTC of 12% (95% CI, 3% to 23%). With sensitivity testing, using this unrealistically high estimate of lifetime risk of MTC (one in 1000), in combination with similarly implausible estimates of allelic heterogeneity (45%) and genetic heterogeneity (40%), the observed frequency of p.Val804Met still only equates to a penetrance for MTC of 46% (95% CI, 1% to 82%).

Studies of detection rates for *RET* mutations vary widely depending on inclusion criteria for individuals/families tested, and variable distributions of different *RET* mutations have been reported from studies of different geographic regions (19–24). Between-study estimates may reflect genuine differences, as a result of disparities in ethnicity and/or founder effects, but may also reflect artifacts from incomplete testing of the full *RET* gene, patient recruitment criteria, and reporting of results. Therefore, we used, for our baseline parameters, estimates based on the largest single center experience reported, in which systematic testing of the relevant exons had been undertaken (19).

The methodology used in the current study does not explicitly take into account age at disease diagnosis or the reduced life expectancy for individuals diagnosed with MTC. It is plausible that a population dataset comprising individuals with common conditions ascertained in middle age would be “de-enriched” for individuals with early-onset, life-limiting disease (survival bias). Likewise, our model assumes that individuals who have developed treatable MTC or have had preventative surgery would be proportionately sampled into the ExAC cohorts compared with the reference population: in practice, it is likely that these individuals would likewise be under-represented (selection bias). However, any such survival bias or selection bias would serve to de-enrich the ExAC cohorts for mutations causative of MTC, only resulting in our calculations of penetrance presented being overestimates. Finally, inclusion in the ExAC cohort would not be influenced by MTC arising later in life (*i.e.*, after inclusion in the whole genome sequencing/whole exome sequencing cohort); hence, later disease would not bias our estimates of penetrance.

Population stratification may cause confounding effects by the skewing of observed allele frequency, for example, as a result of ethnic population-specific effects from founder mutations. The broad makeup of the ExAC series from several outbred subpopulations makes this unlikely. Furthermore, the elevated frequency of p.Val804Met is observed across the four major subpopulations of ExAC in this study. In addition, there is consistency of MAF between ExAC and the larger recently released Genome Aggregation Database dataset, negating a substantial contribution from “chance” sampling variation (type 1 error).

p.Val804Met is one of the most commonly reported pathogenic mutations in *RET.* However, the literature, to date, for p.Val804Met is mixed regarding phenotypic correlates, as well as overall and age-related penetrance. Most typically associated with the FMTC spectrum, there are reports of p.Val804Met associated with pheochromocytomatous but also papillary thyroid cancer. There are reports in the literature of p.Val804Met detected in high-penetrance families, as well as very early-onset cases (25–27). A number of studies have reported C cell hyperplasia as a ubiquitous finding in prophylactically removed thyroid glands from “unaffected” carriers of p.Val804Met (25–27). The only numeric estimate for penetrance for p.Val804Met published to date has been from Rich *et al.* (28), who report penetrance of *RET* p.Val804Met for MTC to age 70 as 87% (95% CI, 71% to 94%), with the median age of detection of MTC at 54 years. This is far and away the largest analysis of its kind published to date, including 160 individuals identified with p.Val804Met via familial cascade genetic screening alone. Therefore, this study would be credited for robustness of methodology, insofar as index cases of MTC were excluded to minimize upward bias in estimates of penetrance. This study confirmed that *RET* p.Val804Met was indeed typically associated with later onset of MTC than typical for other pathogenic germline *RET* mutations but asserted that albeit of later onset, penetrance for disease was nevertheless near complete (28). Therefore, this analysis was seen to be consistent with several earlier descriptive reports in the literature, suggesting that *RET* p.Val804Met must have a different penetrance profile for MTC compared with the other *RET* mutations that typically cause MTC in childhood (26, 29).

Occurrence of two germline *RET* mutations in tandem on the same allele have been described, with p.Val804Met reported in tandem with p.Gln781Arg, p.Glu805Lys, p.Tyr806Cys, or p.Ser904Cys (30). Such tandem genotypes have been demonstrated *in vitro* as having a higher transforming ability compared with their singleton counterparts (31). Commensurate with this, these tandem genotypes are associated clinically with more aggressive early-onset MTC and were assigned highest risk designation by the ATA. Notably, the two-tier/single-exon approach to mutational analysis, previously used in clinical practice and earlier studies, could have been at risk for failure to detect a second mutation in a different exon (*e.g.*, p.Ser904Cys). This raises the possibility that occasional individuals/families with a tandem genotype (and accordingly, more severe phenotype) may have erroneously been ascribed as just having p.Val804Met. This could potentially contribute (likely in small part) to explaining some of the apparently variable penetrance and expressivity that has been widely reported in the literature for p.Val804Met (25, 26). Of note, none of the “second” mutations constituting these tandem genotypes were detected in our population dataset; therefore, consideration of tandem genotypes is not relevant to the penetrance estimates that we present here for p.Val804Met.

Interestingly, homozygosity for p.Val804Met has been detected in individuals undergoing *RET* testing for seemingly sporadic MTC (32, 33), an observation entirely consistent with the comparatively high-population frequency for p.Val804Met that we describe.

The history in the literature, combined with the data we present here, demonstrates beautifully the influence of ascertainment bias on the published estimates of penetrance with which we currently manage our patients. Penetrance for MTC of *RET* p.Val804Met is likely substantially influenced by additional modifier factors, the “dose” of which is distributed across the population. Early testing of *RET* was typically focused on probands with more severe phenotypes (MEN2A or MEN2B), young-onset MTC, and/or a family history of disease. Hence, such analyses would generate estimates reflecting the “combined” penetrance of p.Val804Met *RET* plus a high dose of modifying factors *ipso facto* present in those with severe and/or familial disease. Even when the *RET* mutations are ascertained from an “unselected” series of MTC, these individuals (and therefore, their close relatives) will necessarily have a dose of modifier factors substantially above the population average. The population data presented here (unbiased in its ascertainment by phenotype) reflect that the distribution of modifying factors across the general population is such that only ∼4% of individuals have a sufficient dose of modifiers, such that they will develop MTC when carrying *RET* p.Val804Met. Observed familial clustering supports existence of p.Val804Met *RET* modifying factors that are “shared” within families. Whereas these factors are most likely to be heritable (genetic), shared environmental (“household”) factors may also be relevant.

The observation in control data of a frequency of p.Val804Met, which is approximately fivefold higher than that of any of the other ATA moderate-risk *RET* mutations, is clear evidence that the penetrance of p.Val804Met must be significantly lower than for other mutations. Indeed, our analyses, based on best estimates of genetic, allelic heterogeneity, and lifetime risk of MTC, suggest that the penetrance for p.Val804Met may be <5%. Increasing affordability and throughput of next-generation sequencing technologies have dramatically expanded the volume and reduced the thresholds for genetic testing; *RET* is included on several multigene cancer panels widely tested in oncology and familial cancer clinics. Furthermore, *RET* is one of the 59 genes for which the American College of Medical Genetics and Genomics recommends reporting as a secondary finding (*i.e.*, reporting in individuals with no relevant history of cancer in whom whole exome sequencing or whole genome sequencing is undertaken for unrelated indications) (34, 35). We are just dipping our feet into the oncoming tidal wave of massive expansion in genomic testing, which will be undertaken in contexts increasingly distant from those classic “high-penetrance” multiplex families—analyses of which underpin our current penetrance data. Prophylactic surgery in childhood or young adulthood can be associated with substantial immediate and long-term morbidity with a requirement for lifelong thyroid replacement therapy (7).

The ATA currently recommends prophylactic thyroidectomy for all *RET* mutation carriers, stating, “…the question is not whether prophylactic thyroidectomy should be performed in patients with hereditary MTC, but at what age?” Whereas prophylactic thyroidectomy in early childhood (<5 years) is recommended unequivocally for children carrying mutations categorized as high and highest risk, the recommendations are less didactic for moderate-risk mutations, such as p.Val804Met. However, because of (1) the challenges of long-term monitoring of serum calcitonin in combination with (2) recognition of MTC as an aggressive cancer, only curable if diagnosed when still intrathyroidal, the ATA currently cautions that “…there are significant risks in delaying surgery in family members who have inherited a mutated *RET* allele, regardless of the patient’s age one must balance the risks of thyroidectomy against the possibility that the thyroidectomy will be incurable if it is delayed” (7).

We would suggest that the overall emphasis within ATA recommendations on thyroidectomy, as being prophylactic (*i.e.*, preceding manifestation of disease) and as ultimately “inevitable” for all *RET* mutation carriers, should be better clarified. Inevitable prophylactic surgery may be a reasonable presumptive model for the carriers of most *RET* mutations who have close relatives affected by disease. However, for individuals with *RET* p.Val804Met, ascertained through population-based testing [*i.e.*, with no (or an only distant) family history of disease], truly prophylactic thyroidectomy is likely inappropriate. Based on the data we present and cognizant of the rapidly expanding context of testing in the new “genomic” era, we recommend urgent review by the ATA. In particular, consideration should be given to (1) p.Val804Met-specific management guidance and (2) clinical guidance by context of ascertainment, including explicit recommendation for surveillance with surgery, reflexive only when there is evidence of emerging disease (36).

## Abbreviations:

ATA: American Thyroid Association
CI: confidence interval
E: expected
ExAC: Exome Aggregation Consortium
FMTC: familial medullary thyroid cancer
MAF: minor allele frequency
MEN2: multiple endocrine neoplasia type 2
MTC: medullary thyroid cancer
O: observed
RET: rearranged during transfection
TCGA: The Cancer Genome Atlas
